# Supplementary material for: Regulators of rDNA array morphology in fission yeast
Source: PLoS Genet. 2024 Jul 5;20(7):e1011331. doi: 10.1371/journal.pgen.1011331 (PMC11253961; doi:10.1371/journal.pgen.1011331)

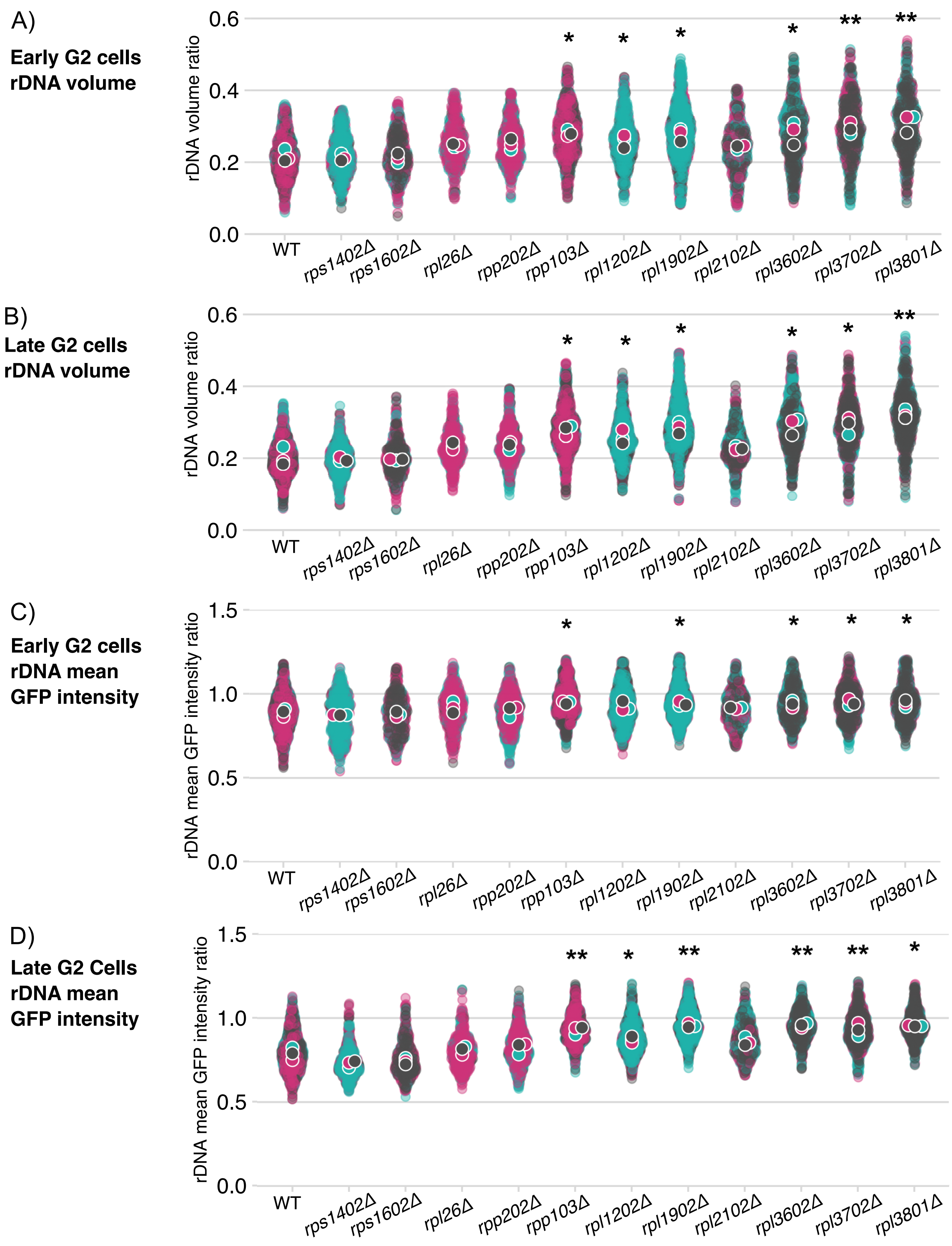
A)
Early G2 cells
rDNA volume
rDNA volume ratio
0.0
0.2
0.4
0.6
WT
rps1402Δ
rps1602Δ
rpl26Δ
rpp202Δ
rpp103Δ
rpl1202Δ
rpl1902Δ
rpl2102Δ
rpl3602Δ
rpl3702Δ
rpl3801Δ
*
*
*
*
**
**
B)
Late G2 cells
rDNA volume
rDNA volume ratio
0.0
0.2
0.4
0.6
WT
rps1402Δ
rps1602Δ
rpl26Δ
rpp202Δ
rpp103Δ
rpl1202Δ
rpl1902Δ
rpl2102Δ
rpl3602Δ
rpl3702Δ
rpl3801Δ
*
*
*
*
*
**
C)
Early G2 cells
rDNA mean
GFP intensity
rDNA mean GFP intensity ratio
0.0
0.5
1.0
1.5
WT
rps1402Δ
rps1602Δ
rpl26Δ
rpp202Δ
rpp103Δ
rpl1202Δ
rpl1902Δ
rpl2102Δ
rpl3602Δ
rpl3702Δ
rpl3801Δ
*
*
*
*
*
D)
Late G2 Cells
rDNA mean
GFP intensity
rDNA mean GFP intensity ratio
0.0
0.5
1.0
1.5
WT
rps1402Δ
rps1602Δ
rpl26Δ
rpp202Δ
rpp103Δ
rpl1202Δ
rpl1902Δ
rpl2102Δ
rpl3602Δ
rpl3702Δ
rpl3801Δ
**
*
**
**
**
*

E)

Early G2 cells
Nucleolar volume

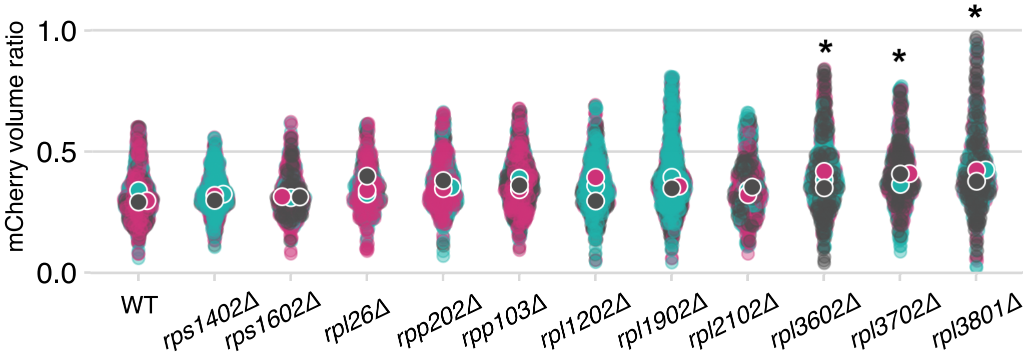

F)

Late G2 cells
Nucleolar volume

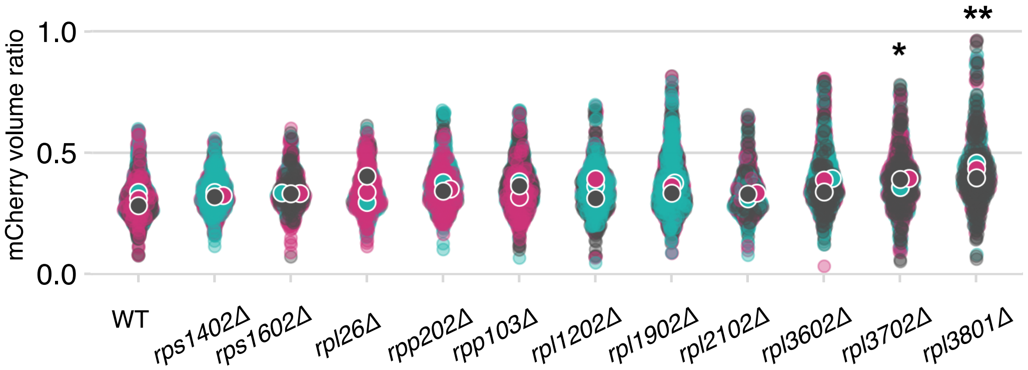

Supplement: S12 Fig — All plots show quantification for cells in early G2 (A-C) or late G2 (D-F) G2. Each plot shows 3 biological replicates per strain, with a distinct color per replicate. Median values for each biological replicate are plotted as larger circles. Statistical significance was determined for each RP mutant relative to WT by unpaired t-test. Statistical significance is noted by an asterisk, where *p<0.05 and **p<0.01. (PDF) [file pgen.1011331.s012.pdf]
