# Supplementary material for: Regulators of rDNA array morphology in fission yeast
Source: PLoS Genet. 2024 Jul 5;20(7):e1011331. doi: 10.1371/journal.pgen.1011331 (PMC11253961; doi:10.1371/journal.pgen.1011331)
Supplement: Attachment — Submitted filename: Review for PLOS Genetics.docx [file pgen.1011331.s025.docx]

This is a highly-sophisticated study of the appearance of the nucleolus in fission yeast. It attempts to identify circumstances that affect the size of rDNA and the nucleolus as well as signaling pathways that mediate their effects.

Although the manuscript tabulates an enormous amount of information, there is no crisp take-home lesson. Ultimately, the authors conclude that perturbation of the nucleolus can require any of several signaling pathways. At first glance this is similar to the classic assertion that “the nucleolus is sensitive to many factors.”

The authors could consider separating out their Omics data. Alternatively, they could present their screen entirely in the Supplementary Data section and use the Results to announce their central observations.

Specific Comments

The Introduction should describe and illustrate the basic organization of rDNA repeats (not just a single repeat) and the chromosome on which they are found.

Also:

1) It may seem obvious, but there is no simple statement that allows the reader to know how the apparent volume of rDNA was measured.
2) There also is no simple statement that allows the reader to know how the volume of the nucleolus was measured.
3) Were both of these parameters normalized to the total volume of the nucleus (as detected with GapR or Hoechst) ? If not, why not ? Normalization of GapR to Hoechst could be useful.

If these measurements depend on the distribution of GapR-GFP, the reader needs to be told – in the Results section –

- how the relevant regions were delineated.
- whether the measurements are influenced by the orientation of the nucleus and how this issue was dealt with.

We are told that “Cells were grown at 30°C for a minimum of 5 hours to allow for GapR-GFP induction.” The constancy of these conditions could be critical for all interpretation of the data. “More than 5 hr” seems to be too vague a statement if we are to pay attention to quantitative measurements.

Since the authors show that expression of the reporters depends on the cell cycle, couldn’t cell cycle perturbation account for their key fluorescent data ?

If these measurements depend on the distribution of Nhp2, we should be told whether this protein is found throughout the nucleolus. We should also be told why it was chosen and what is know about the NHP2 promoter..

Contrary to the authors claim, this reader cannot see evidence that actinomycin D causes Nhp2 to diffuse throughout the nucleus (The figure shows only a qualitative very partial effect that is scarcely in agreement with the primary data). Despite statements to the contrary, I find no suggestion of nucleolar “cap” formation.

Judging from Figure 3B, the deletions that increase or decrease GFP intensity have only a modest effect (<20% increase or decrease). The impact on rDNA volume seems larger. In any event, both axes in Figure 3B should start at zero values.

We are told that some strains had equivalent 28S copy number. Where in the Methods section is the procedure for making these estimates ?? And how accurate are they ? All I see is a mention of using digital droplet PCR.

The described resistance of RPL mutants to a TOR inhibitor (Figure 5A) seems not to be shared by all mutants. What are the distinguishing characteristics of those that are resistant ? Are the ribosomal proteins in question all playing similar roles in subunit production ?

Please explain why the term “overtwisted” is used instead of “super-coiled.”
