# Supplementary material for: Regulators of rDNA array morphology in fission yeast
Source: PLoS Genet. 2024 Jul 5;20(7):e1011331. doi: 10.1371/journal.pgen.1011331 (PMC11253961; doi:10.1371/journal.pgen.1011331)
Supplement: Attachment — Submitted filename: 2024-02-08-GapR-manuscript-reviewer-responses-FINAL.docx [file pgen.1011331.s026.docx]

Dear Dr Gerton,

Dear Jen,

Your work entitled 'Regulators of rDNA morphology in fission yeast'  submitted to PLoS Genetics has now been carefully evaluated by four experts in the field of ribosome biogenesis. I have also read the paper with interest.

While all four referees, and myself, recognize the timeliness and originality (use of an heterologous reporter, live cells, etc.) of your approach and the importance of the topic (how nucleolar morphology reflects function), they all raised substantial concerns that would need to be fully addressed in a revised version of the manuscript.

Personally, I also found the approach very original, but I agree that to prove that GapR-GFP does not interfere with Pol I function, there is a need to perform a pulse-chase/metabolic labelling (or EM spreads). Also, I often thought, is Nhp2 really the best marker here? Could some results be confirmed with another nucleolar protein co-staining? Would it be possible to confirm the change in rDNA organization by use of an endogenous protein detection (eg an rDNA binder) or by some other means?

It will be important also to explain well how rDNA and nucleolar volume were calculated, and how the effects of cell-cycle were taken into account (were cells synchronized?). A few excellent recommendations on semantics were suggested, which will also improve the paper.

Based on their overall positive assessment and constructive criticisms, I would like to suggest you prepare a revision of your manuscript. Since this will require the addition of novel experimental work, I would like to suggest an initial 6-month deadline for resubmission. Feel free to contact me if you need to discuss your revision plan, or if you need an extension.

Best wishes,

Denis

We thank the reviewers and Editor for their attention and thoughtful comments on how to improve the manuscript. In brief, we have now included new experiments, new figures, and text edits, summarized below. We have also included a point by point response.

1. We now provide evidence that the induction of GapR-GFP does not interfere with the production of rRNA via a pulse labeling experiment (Fig S2) and we provide an experiment to address the specificity of GapR binding using a GapR mutant (Figure 1).
2. We have visualized and confirmed the changes in rDNA morphology in glucose starvation and an RP mutant using a second marker, namely a subunit of RNAP1 labeled with BFP (Figures S8 and S13), and further explain the choice of Nhp2 as a nucleolar marker.
3. For many experiments we now also compare only cells in G2 (Figures 3, 4, 6, and supplemental figures S2, S4, S6, S7, S8, S9, S12, S13, and S15).
4. We provide rRNA pulse labeling results for an RPL mutant (S14).
5. We expanded and improved the explanations of our imaging quantification methods and analysis.
6. We edited the manuscript to use RPL and RPS terminology instead of 40S and 60S.
7. We toned down claims regarding ribosome biogenesis and transcriptional activity and revised the model (Fig 8 model and throughout).
8. We reordered and edited the manuscript to better focus on the strengths of the work. This included the addition of many supporting figures and the removal of 2 experiments related to the Pmk1/RP double mutant phentoype.

We wanted to provide the Editor and reviewers with additional information regarding the request for a second marker for rDNA. This turned out to be extremely challenging. In brief, we tried tagging five different rDNA associated proteins under a constitutive promoter on an integrating plasmid (Hmo1, Rpa43, Nuc1, Rrn10, Uaf30). We only recovered viable cells with nucleolar localization for Rpa43, suggesting that introducing tags on rDNA binding proteins is poorly tolerated even with the endogenous gene intact. Furthermore, the BFP tag both bleached rapidly and caused phototoxicity, preventing us using it for robust timelapse imaging. As an alternative, we tried to visualize the rDNA arrays using a Hoescht intercalating dye in live cells. The conditions that allowed the dye to penetrate the cells and mark the rDNA arrays were inconsistent and not compatible with our experiments. This combination of difficulties helps to highlight that using an exogenous protein that binds to the intergenic region of the rDNA repeats as a live marker is an innovation.

This is a highly-sophisticated study of the appearance of the nucleolus in fission yeast. It attempts to identify circumstances that affect the size of rDNA and the nucleolus as well as signaling pathways that mediate their effects.

Although the manuscript tabulates an enormous amount of information, there is no crisp take-home lesson. Ultimately, the authors conclude that perturbation of the nucleolus can require any of several signaling pathways. At first glance this is similar to the classic assertion that “the nucleolus is sensitive to many factors.”

The authors could consider separating out their Omics data. Alternatively, they could present their screen entirely in the Supplementary Data section and use the Results to announce their central observations.

Specific Comments

The Introduction should describe and illustrate the basic organization of rDNA repeats (not just a single repeat) and the chromosome on which they are found.

Good point, additional description of the fission yeast rDNA repeat organization is included in the results section, lines 157-159, page 8.

Also:

1) It may seem obvious, but there is no simple statement that allows the reader to know how the apparent volume of rDNA was measured.
2) There also is no simple statement that allows the reader to know how the volume of the nucleolus was measured.
3) Were both of these parameters normalized to the total volume of the nucleus (as detected with GapR or Hoechst) ? If not, why not ? Normalization of GapR to Hoechst could be useful.

A more detailed description of how nuclear and nucleolar parameters were calculated, as well as how nucleolar values were normalized to the nucleus, is now included in the results section on page 11 lines 226-240, as well as in the methods section page 32, lines 687-696. Our attempts to use Hoechst for normalization were unsuccessful because the staining was unreliable and compromised cell health.

If these measurements depend on the distribution of GapR-GFP, the reader needs to be told – in the Results section –

- how the relevant regions were delineated.
  - This is now described in more detail in the methods section, lines 687-696 on page 32.
- whether the measurements are influenced by the orientation of the nucleus and how this issue was dealt with.
  - This is now described in lines 681-683 of the methods section, page 32.

We are told that “Cells were grown at 30°C for a minimum of 5 hours to allow for GapR-GFP induction.” The constancy of these conditions could be critical for all interpretation of the data. “More than 5 hr” seems to be too vague a statement if we are to pay attention to quantitative measurements.

A more specific description of growth conditions – specifically, that growth was done for 5-6 hours - is now included in line 629 on page 29 in the methods section.

Since the authors show that expression of the reporters depends on the cell cycle, couldn’t cell cycle perturbation account for their key fluorescent data ?

This is an excellent suggestion. We now account for cell cycle by limiting our analysis to cells in G2. Furthermore, we breakdown our analysis to examine cells in early or late G2 using cell length as a proxy for time in G2. Please see lines 266-268 in the results section on page 13 and lines 683-687 on page 32 in the methods section for information on how we limited our analysis. The trends we observed in asynchronous cells remained overall consistent.

If these measurements depend on the distribution of Nhp2, we should be told whether this protein is found throughout the nucleolus. We should also be told why it was chosen and what is know about the NHP2 promoter..

We address the distribution of Nhp2 in lines 193-195 on page 9. Nhp2 was chosen because it is an rRNA processing protein that is found in the nucleolar compartment throughout all cell cycle stages including mitosis. While the promoter is not well studied, we are able to visualize the nucleolus at all cell cycle stages using Nhp2 (see Fig 2). This marker also has precedence since it has been successfully used to identify fission yeast nucleoli in other studies (see Jones and Forsburg 2023). Furthermore, we found that compared to other established fission yeast nucleolar markers such as Gar2, fluorescently tagging Nhp2 had no measurable effect on cell growth.

Contrary to the authors claim, this reader cannot see evidence that actinomycin D causes Nhp2 to diffuse throughout the nucleus (The figure shows only a qualitative very partial effect that is scarcely in agreement with the primary data). Despite statements to the contrary, I find no suggestion of nucleolar “cap” formation.

The wording in lines 299-301 on page 14 have been changed to address this comment (now Fig S7). We describe and demonstrate the nuclear dispersion of Nhp2 but emphasize that most of the Nhp2 signal remains nucleolar. In lines 303-307, we discuss the parallels between our observation that rDNA loci in fission yeast condense and relocate to the nucleolar periphery when RNA pol I is inhibited, similar to the rDNA arrays in mammalian cells. We comment that rDNA reorganization under stress is conserved, but we do not refer to the condensed fission yeast rDNA foci as “caps” which is a term used in mammalian cells.

Judging from Figure 3B, the deletions that increase or decrease GFP intensity have only a modest effect (<20% increase or decrease). The impact on rDNA volume seems larger. In any event, both axes in Figure 3B should start at zero values.

Changing the X-axis to zero creates a significant amount of white space in the figure, as we do not see rDNA volumes below 0.6 (now Fig 5). While we could scale these axes to make them both start at zero, we feel that leaving the true values is more representative of the data.

We are told that some strains had equivalent 28S copy number. Where in the Methods section is the procedure for making these estimates ?? And how accurate are they ? All I see is a mention of using digital droplet PCR.

The information describing the method and associated error has been added to the methods section, lines 858-860 on page 39. Digital droplet PCR is a highly sensitive and quantitative assay for measuring copy number (see Salim et al 2017 PloS Genetics).

The described resistance of RPL mutants to a TOR inhibitor (Figure 5A) seems not to be shared by all mutants. What are the distinguishing characteristics of those that are resistant ? Are the ribosomal proteins in question all playing similar roles in subunit production ?

We agree this is a point of uncertainty, and not one we have been able to resolve. Additional descriptions of the RPL TOR inhibitor resistance phenotype have been added to the results in lines 448-452 on page 21. We observe a rough correlation between the strength of the rDNA volume and intensity phenotypes and resistance to the TOR inhibitor in the RP mutants. We acknowledge that the variation in both rDNA and Torin1 resistance phenotypes may become clearer as the canonical and moonlighting functions as well as expression of individual RP paralogs is better characterized in fission yeast.

Please explain why the term “overtwisted” is used instead of “super-coiled.”

We have added additional clarification in the manuscript to better explain the term (lines 184-186 on page 9). "Overtwisted” is a specific term that describes the preferred form of DNA that GapR binds. Overtwisted DNA has a smaller diameter relative to B-form DNA and results from torsional stress introduced by upstream polymerases as they unwind the DNA helix. “Super-coiled” can refer to positive or negative coiling. Therefore, “overtwisted” is a more precise word choice.

Reviewer's Responses to Questions

**Comments to the Authors:
Please note here if the review is uploaded as an attachment.**

Reviewer #1: referee#1's report was posted as an attachment and should be downloaded

Reviewer #2: The manuscript entitled " Regulators of rDNA morphology in fission yeast" by Cockrell et al., explore the ribosomal DNA structure using a bacterial protein, GapR, known to associate with overtwisted DNA. Authors characterize GapR binding in vivo by quantitative imaging, and could show that GapR can be used as a very useful reporter construct to investigate rDNA structure in vivo. rDNA modification during cell cycle, upon transcriptional repression, or TOR deregulation is of interest. Using this new reporter, they were able to screen an entire library of haploid deletion, which is clearly a master piece of this work. Due to frequent variation of rDNA copy number which could affect screen results, authors focused on 118 candidates to validate screen results. They could identify a very interesting effect of 60S biogenesis defect on rDNA morphology

The following part of the manuscript is less focused, with some connections with signaling pathway which are not always conclusive. Some results presented here appears essentially preliminar, such as Synthetic Genetic Array to explore signalling pathways altered in 60S mutants (presented in figure 5B). Global interaction presented here are not directly demonstrating authors statement (Our genetic screen indicated all assayed signaling pathways can provide input into Torin1 resistance). Figure 6 depict a possible connection with Cell Integrity MAPK pathway which appears more robust.

Overall, this study is very interesting and provide some important insight on rDNA structure in vivo. Some important control are required, and extensive editing is required. Numerous data are presented the text, in figures and supplementary figure that are not always crucial (see SGA screen). Authors should clarify the text, and select required figures.


Major critique:

GapR expression has no obvious negatively impact on rDNA function or cell health. Authors should also check that rDNA copy number is not impacted using either qPCR, or Pulse-field gel electrophoresis (or digital droplet PCR as in figure 1C).

Copy number was checked by ddPCR during the initial strain construction and strains with similar copy number were selected. The 28S copy number for each strain is listed in table S6. For the induction experiments, GapR-GFP expression was induced for 5-6 hours, which represents ~2 generations in fission yeast, a timeframe that is insufficient for significant changes in copy number.

What about the ratio transcribed / silenced genes in rDNA of . pombe ? When the number of genes increase, does the ratio of active genes/silenced rDNA change? This analysis using psoralen cross-linking would be useful to explore if GapR binding is specific of transcribed genes.

This manuscript presents GapR-GFP as a unique tool to investigate rDNA organization. The relationship between GapR-GFP binding and the activity of individual rRNA genes is interesting but very difficult to address. Psoralen crosslinking is a bulk method and would not allow us to directly relate binding and activity at individual gene. This type of single gene analysis will likely require long read sequencing methods or immunoEM that are outside the scope of this manuscript but would certainly be interesting to explore in the future.

Authors showed that GapR expression does not affect accumulation of rRNA or pre-rRNA (S2A). However, accumulation of rRNA can be equally affected by transcription, transcript stability and maturation rate. Authors should perform pulse labelling experiment to establish that in vivo transcription is not affected by GapR expression. Pulse labelling with 5mM 4-thiouracil is described in the method section, but is revealed using total RNA detection. In such detection, it is unclear which RNA species is detected. Why not using such thiouracil labelled RNA and analyze pre-rRNA, or rRNA labelled during such 2 min using immobilized probe, similar to Run-on assay (for budding yeast, see wery et al., RNA, 2009).

To examine if GapR expression affects rRNA transcription, we applied the 4-TU pulse-labelled spot assay to a strain expressing GapR-GFP and a control strain expressing GFP-NLS (see Fig S2). We observed no significant differences in nascent transcript levels. While our approach does detect total RNA, Udem and Warner demonstrated in 1972 that the vast majority of RNA being produced during a 5-minute pulse is rRNA. Furthermore, they demonstrated that a 5-minute pulse produces a similar overview of rRNA products as a 1-minute pulse.

Minor critique:

In introduction, known re-organization of rDNA along cell cycle should be mention, to introduce the notion that rDNA reorganization depend for sure of transcriptional activity but also on cell cycle.

We briefly describe the dynamic organization of the rDNA during the budding yeast cell cycle in the introduction, see lines 141-142 on page 7.

For imaging, wide field micrography to see the distribution of patterns of GapR-GFP among a yeast population would be very usefull.

The images in Figure 2 provide an overview of GapR-GFP localization throughout the WT cell cycle.

GapR(1-76) fuse to GFP should be shown as comparison to the very clear rDNA binding of full-length GapR.

Thank you for this suggestion. Images of the truncated GapR(1-76)-GFP strain have been added to Figure 1 to demonstrate that full-length GapR is needed to robustly detect the rDNA arrays.

In budding yeast rRNA production is decreased during mitosis. Here, authors observed GapR binding in mitosis. What about the transcription level in S.pombe mitosis ?

GapR is likely an imperfect reporter for rapid changes in transcriptional activity during mitosis because once bound, it is very stably associated with DNA. Using FRAP (Fig S3), we demonstrate that GapR-GFP turnover does not occur over a 5 minute timeframe. This finding is supported by GapR-GFP’s binding activity in its native organism – *C. crescentus –* where GapR-GFP loading only occurs during DNA replication. While rRNA transcription is likely decreased during mitosis in pombe, GapR-GFP binding is stable and not likely to be affected by decreased transcription in a single cell cycle.

Figure S5 is unclear and should be corrected; panel A is not informative, panel B is only showing total RNA, and not a transcriptional shut-off with 2 hours of glucose starvation (S5 Fig). (Please correct spelling in figure legend biontinylated RNA).

Thank you. Additional figure labeling and description in the figure legend is now included.

Figure S6. Authors should rephrase interpretation “Actinomycin D treatment causes Nhp2 diffusion throughout the nucleus (S6A Fig)”. An increase nucleoplasmic signal is observed, but a very large fraction of Nhp2 remains nucleolar. This should be mentioned.

The wording in lines 299-302 on page 14 have been changed to address this comment (now Fig S7). We describe the increase in nuclear signal but emphasize that most of the Nhp2 signal remains nucleolar.

In the screen, some expected hits are present (SPBC11G11.05, or sp-Rpa34) but other such as sp-Hmo1 (hmo1 / SPBC28F2.11) are not present in the table. This is surprising. Why in figure 3C, gene SPAC607.02c is named SPAC607.02c.1 ? Authors should mention which mutant is present, or absent from the deletion collection analyzed here.

Table S1 includes all mutants that were included in the imaging screen and had more than 30 cells for quantification; this table includes hmo1, which was not identified as having a significant rDNA phenotype. Table S3 (table S2 in the original manuscript) lists the strains that were considered “high hits” from the initial screen. A list of strains that were excluded from this analysis, either due to having less than 30 cells or failure to survive generation of the GapR-GFP screen library, is now included as Table S2. The hmo1 mutant was included in the initial imaging screen and is present in table S1. Mutants like hmo1 may have rDNA phenotypes, however there are several factors that influence our ability to detect these phenotypes. First, rDNA repeat copy number variation is an unavoidable contributing factor in such large-scale screens. While we were able to account for rDNA copy number variation during subsequent validation of selected screen hits, we were not able to control for variability in rDNA copy number during generation of the initial screening library. Second, we used a stringent threshold for rDNA volume and rDNA mean GFP intensity values to identify screen hits. This strict cutoff may have caused us to miss weaker, though still significant, mutants with rDNA phenotypes. Finally, the Bioneer deletion collection contains incorrect strains where the listed gene deletion is not present. Our selection for mutants with stronger phenotypes, the variability in rDNA repeat copy number, and limitations with the validity of the Bioneer deletion collection are all possible explanations for why some expected mutants were not identified as having rDNA phenotypes in our screen. These limitations of our imaging screen have now been included in the text, lines 367-373 on page 17.

Some results are confusing (While we observed a trend towards increased Pmk1 protein levels, this trend was not statistically significant (S11 Fig)). If not conclusive, this should not be shown. Alternatively, experiments should be repeated to strengthen the tendency authors discuss here.

Thank you for this suggestion, and we apologize for not making this section of the manuscript clearer. To focus the manuscript on our characterization of rDNA spatial organization using GapR-GFP, this experiment was removed.

Some small editing are required (“5/12/2023 4:22:00 PM” present page 6 lane 114)

Careful references editing should be made for example:

in ref 3, journal is missing : (Derenzini M, Trere D, Pession A, Montanaro L. Nucleolar Function and Size in Cancer Cells. 804 1998;152:7 )

in ref 19, a copy/paste when wrong, Berger, Barkai and editors are not authors of this work. (Guo MS, Kawamura R, Littlehale ML, Marko JF, Laub MT. High-resolution, genome-wide mapping of positive supercoiling in chromosomes. Berger JM, Barkai N, editors. eLife. 2021;10:e67236. )

Thankyou for noticing these errors made by our reference manager, we have now manually edited them.

Reviewer #3: This ms submitted to PLoS Genetics by Cockrell et al entitled “Regulators of rDNA morphology in fission yeast” takes a unique approach to discovering factors required for maintaining the rDNA arrays in a yeast that divides, S. pombe. They have pioneered the use of the GapR protein from Caulobacter that binds to twisted DNA, including in the rDNA repeats, to delineate the spatial organization of the rDNA in live cells. GapR was modified with S. pombe codon optimization, fused to GFP for detection, and placed under the control of an estradiol promoter in fission yeast. The basic assays that they use to report rDNA spatial organization are rDNA volume (from Nhp2-Cherry) and rDNA mean GFP intensity (from GapR-GFP). They screened for nonessential genes that when deleted affected these parameters and found many RPL proteins. Surprisingly, the observed phenotype, based on the GapR-GFP signal, was consistent with increased ribosome biogenesis. To define the signalling pathways that contribute to this, they used Synthetic Genetic Arrays and found that RPL insufficiency results in activation of the Pmk1 pathway. This study is an exciting approach, carefully done, that combines microscopy with genetics in S. pombe to probe ribosome biogenesis, revealing new signalling pathway inputs.

Thank you for this positive evaluation of our work.

At the same time, I think there are some things that could be improved prior to publication.

1. The paper unfortunately relies on inference for some of the major conclusions and could be strengthened by some simple biochemical experiments. For example, increased ribosome biogenesis in the delta RPL strains is not tested directly but could be. Furthermore, decreased RPL levels is not tested at the level of protein, but could be. It would be important to know if these biochemical results concur with the microscopy, especially since this is the first use of this reporter gene.

We agree with the reviewer regarding the strengths and weaknesses of our study. Our study relies heavily on genetics and microscopy. To partly address this request and those of other reviewers and the editor, we performed an additional measurement of nascent transcripts in an RPL mutant using the 4-thiouracil nascent RNA spot assay (see Fig S14). We did not observe a significant increase in nascent RNAs in this RPL mutant. Although we think GapR-GFP is an excellent marker of the rDNA, it may be an imperfect reporter of transcriptional activity because once bound, it is very stably associated with DNA (FRAP result Fig S3). Moreover, small changes in the production of nascent rRNA may be difficult to detect reliably. We agree that there are more experiments that could be performed, such as the examination of ribosomal protein levels (although we did include analysis of mRNA levels in the supplement) and measurements of ribosome biogenesis. We chose to focus our revisions around the guidance provided by the editor, and we toned down our claims regarding how rDNA morphology as detected by GapR-GFP directly relates to transcription and ribosome biogenesis.

2. There is a prevalent use of jargon that renders the ms difficult to understand. These are some examples where jargon is used and muddles the meaning. “60S ribosome subunit gene deletion” “our screen identified 60S mutants” “60S hits” It is all through the ms. I do not think that the RPL mutations should be described in this way. “60S ribosome subunit gene deletion” would mean to me deletion of the large subunit rRNAs, not the large ribosomal subunit proteins. A better way to say “our screen identified 60S mutants” is to say “our screen identified mutations in proteins of the large ribosomal subunit.” “60S hits” should be “RPL hits”. It is critical to avoid the use of shorthand, confusing jargon and refer to the RPL protein themselves, after first defining them as protein components of the large ribosomal subunit.

To address this comment and reduce reader confusion, “60S” has been changed to “RPL”, and “40S” has been changed to “RPS” throughout the manuscript. These terms are defined in the introduction (lines 167-168 page 8) for RPL mutants and in the results section (line 383 page 18) for the RPS mutants.

3. I must not understand the results in Fig 5, as I see variable torin resistance among the strains. Please state what was used as criteria with each concentration. For 10 microM, perhaps any growth in the 3rd dilution counted as resistance? If you instead used growth in all 5 dilutions, only one would be resistant. Perhaps I missed this logic, please explain.

Additional description was added to the figure caption to address this confusion (now Figure 7). Resistance was defined as mutant growth compared to WT growth. For example, WT growth was observed in the 3^rd^ column with 10um torin1, and mutant growth was observed in the 4^th^ and 5^th^ columns. This indicates that the mutant is able to grow better in the presence of 10um torin1 than the WT strain, demonstrating torin1 resistance. We also now explicitly mention the variation in Torin resistance and add that “the variation in phenotype may become clearer as the canonical and moonlighting functions as well as expression of individual RP paralogs is better characterized in fission yeast” on page 21 lines 450-452.

4. While the ms relies on live cell signals from two reporter genes, a calculation is made in some of the figures that is related to them but is not defined that I can see. What is the rDNA volume ratio vs the rDNA volume? I don’t understand the ratio part—ratio of what? Please define and put into the text and methods. This can be clarified in the text.

A more detailed description of how nuclear and nucleolar parameters were calculated, as well as how nucleolar values were normalized to the nucleus, is now included in the results section on page 11 lines 227-240, as well as in the methods section page 32, lines 687-696.

5. The Discussion could be improved with greater attention to the published details. I do not agree with the interpretation that the haploinsufficiency of the bone marrow failure syndrome ribosomopathies acts as the oncogenic trigger. Indeed, because these RPS and RPL mutations and deletions cause bone marrow failure, they have reduced ribosome biogenesis, not activated as is found here. Furthermore, ongoing longitudinal studies on patient cells is revealing mutant p53 and oncogene activation as the causes for cancer predisposition.

This is an excellent point that encouraged us to modify and clarify this section of the discussion. The analogy we are making to the RP deletions in fission yeast is that the haploinsufficiency for RP genes may lead to compensatory changes that in the long term select for proliferation and eventually oncogenic progression. The discussion has been modified on page 26 and includes the below reference (PMC406397) suggested by the reviewer.

6. Also, perhaps the Discussion could be strengthened by comparing the results here to the work of Nancy Hopkins in zebrafish PMC406397.

Typos: Line 114. Irrelevant writing.
Line 583: “Septation index” should be bolded. The septation index experiment has been removed.

Reviewer #4: Synthesis of ribosomes is a major energy consuming process in the cell and is tightly regulated according to different physiological conditions. Thus, it is discussed that cells employ different pathways to ensure a balanced up- and downregulation of the structural ribosomal components. One of the first steps of ribosome biogenesis is ribosomal (r)DNA transcription producing rRNA from multicopy tandemly repeated genes in a special nuclear compartment, the nucleolus. In the nucleolus the rRNA undergoes co-transcriptional folding and processing, and it is known that nucleolar structures change in good correlation with the activity of the rRNA synthesizing and maturing machineries. Clearly, a deeper understanding of the interrelationship between rDNA morphology and the regulation of ribosome biogenesis and signaling pathways is lacking.

In this work, the authors express the bacterial DNA-binding protein GapR in fusion with GFP in fission yeast as a marker for both spatial rDNA organization and increased rDNA condensation. GapR was previously shown to bind preferentially to transcriptionally induced overtwisted DNA. Accordingly, GapR-GFP binding should reflect changes in rDNA topology which might be provoked by altered Pol I transcription. The GapR marker protein was used to screen for mutants leading to alterations in rDNA topology. Interestingly, many 60S ribosomal protein gene deletion mutants showed changes in GapR binding. Surprisingly, some of the mutants were resistant against treatment with an inhibitor of the TOR pathway, Torin 1. The Torin 1 resistance phenotype was used to identify signaling pathways which might be responsible for the compensation of the r-protein gene deletion. The authors suggest that the MAP kinase Pmk1 which is known to respond to environmental stress and upregulates ribosome biogenesis triggers the Cell Integrity MAPK pathway when cells suffer from insufficient expression of ribosomal proteins.

Overall, this is an interesting study which investigates a possible connection between the regulation of spatial rDNA organization, ribosome biogenesis and signaling pathways. However, to support the conclusions some aspects should be further experimentally addressed, and several ambiguities must be clarified before publication.

Major problems
1) Most of the experiments shown in the study rely on the DNA-binding protein GapR-GFP which was shown to bind preferentially to transcriptionally overtwisted DNA. In the present study GapR-GFP is employed as sensor to detect transcribed rDNA. However, it cannot be excluded that signal intensity is also due to non-specific GapR DNA-binding. For instance, AT-content of the DNA is one determinant of GapR binding. Furthermore, reduction of ribosomal protein expression could result in defects of co-transcriptional ribosome assembly, which in consequence may lead to defects in pre-rRNA processing or to R-loop formation within the rDNA. Thus, altered DNA or RNA structures might potentially influence GapR binding. Therefore, it is important to include proof-of-principle experiments like FISH to strengthen the argument that GapR-GFP indeed visualizes possible changes in rDNA topology.

We performed two experiments to address these concerns. First, we include data in Figure 1 from a mutant version of GapR that binds DNA nonspecifically and show that it is unable to highlight the rDNA arrays. Second, we compared the features of GapR binding to a second marker, Rpa43-BFP, a subunit of RNA pol I in a WT and RPL mutant strain and in glucose starvation. We observed a similar increase in rDNA volume in an RPL mutant strain(Fig S13). In glucose starvation we observed a similar condensation phenotype (Fig S8). These findings indicate that the rDNA spatial organization phenotypes we identified with GapR-GFP can be reproduced with an alternative native protein and are not apparent with a mutant version of GapR, lending confidence to our observations and interpretations.

2) Apparently, GapR recognizes especially overtwisted DNA. It is questionable whether GapR is always a suitable tool to analyse expansion and condensation of rDNA since it might dissociate from the DNA if the DNA is not anymore overtwisted, but still expanded. This could be investigated by comparing GapR-rDNA interaction with the rDNA association of a bona fide rDNA-binding protein.

We agree that GapR is likely an imperfect reporter of rapid changes in transcriptional activity in a single cell cycle because once bound, it is very stably associated with DNA. Using FRAP (Fig S3), we demonstrate that GapR-GFP turnover does not occur in a 5 minute timeframe. This finding is supported by the binding activity of GapR in its native organism – *C. crescentus –* where loading only occurs during DNA replication and appears stable throughout the cell cycle. We found that Rpa43-BFP shows poor association with the rDNA in glucose starvation, highlighting GapR’s ability to stay bound to DNA even when other transcription-dependent factors are unable to stay bound (Fig S8).

3) The authors claim that rDNA morphology is dynamic and reflects ribosome biogenesis activity. It is necessary to correlate changes in rDNA morphology with changes in ribosome production. The impact of nutrient depletion on 40S/60S pre-ribosome production (e.g., in polysome profiles) and especially on Pol I-dependent transcription (e.g. pulse-chase experiments, ChIP with Pol I-specific transcription factors) should be correlated with the GapR readout in a time dependent manner.

We toned down our claims regarding how rDNA morphology as detected by GapR-GFP directly relates to transcription and ribosome biogenesis, but more simply as a tool to visualize organization. It will require experiments beyond the scope of this manuscript to demonstrate more precisely the nature of the correlation between rDNA morphology and ribosome biogenesis processes. To partly address these comments, we examined nascent transcripts in RPL mutants using the 4-thiouracil nascent RNA spot assay (see Fig S14). We did not observe a significant increase in nascent RNAs in RPL mutants. Therefore, the morphological changes observed via GapR do not necessarily correlate with transcriptional activity. Furthermore, we assayed binding of GapR by ChIP in the RP mutants and show that it is only very mildly altered, consistent with the findings from the transcriptional pulse labeling assay. We also used a subunit of RNA Pol I as a second visual marker for GapR to confirm the observed morphological changes in rDNA over the cell cycle and in the RP mutants. All of these experiments help to clarify and strengthen our claims.

4) The result that haploinsufficiency of some but not all r-proteins counteracts TOR inhibition is surprising especially since it was shown that the TOR pathway regulates ribosome synthesis through r-protein (RP) and ribosome biogenesis (Ribi) regulons. Furthermore, inhibition of the TOR pathway by Torin 1 blocks also tRNA synthesis by reduction of Maf1 phosphorylation which should also lead to growth defects. Indirect effects how r-protein haploinsufficiency might compromise Tor inactivation should be excluded. Therefore, it is necessary to show how the presence of Torin 1 influences rDNA transcription and ribosome biogenesis both in the mutants which were resistant to Torin 1 and in those which were not Torin 1 resistant. ChIP using rDNA- associated Pol I transcription factors, pulse-chase analysis and polysome profiles might be helpful tools to solve these issues.

We speculate that the RPL mutant resistance to TOR inhibitors results from overactivation of the TOR pathway and/or related pathways that can promote ribosome biogenesis, such as the newly discovered Pmk1. We do not currently understand the molecular mechanisms underlying Torin1 resistance in some RP mutants and not others. We now acknowledge in the manuscript that the variation between mutants is worthy of further investigation and can only be resolved when we have a better understanding of the canonical and moonlighting functions as well as expression of individual RP paralogs, but we feel this is beyond the scope of this manuscript.

5) The synthetic effect observed in cells which are haploinsufficient for distinct 60 S r-proteins and carry a deletion of the PMK1 gene should be better characterized. In these strains, the spatial organization of rDNA and ribosome biogenesis, rDNA transcription and pre-ribosomal assembly should be analyzed (see point 4).

Thank you for this suggestion. We agree that the connection between pmk1 and RPL gene deletion should be further characterized, now noted in the manuscript. Given time constraints and our focus on revision suggestions made by the editor, we decided to remove the section of the manuscript with the Western blot and septation index.


Minor problems

1) S2 Fig. To analyze the effect of GapR-GFP expression on rDNA function, pulse-chase (e.g. thio-uracil labeling) experiments are more meaningful since analysis of steady state transcripts reflects both rRNA synthesis, rRNA processing and rRNA degradation.

To examine if GapR expression affects rRNA transcription, we applied the 4-TU pulse-labelled spot assay to a strain expressing GapR-GFP and a control strain expressing GFP-NLS (Fig S2). We did not observe any significant changes in nascent transcript levels due to GapR-GFP induction. We have now provided an analysis of both steady-state and nascent rRNA transcripts in GapR-GFP expressing cells, both arguing that GapR does not affect rRNA transcription.

2) Can the authors rule out that r-protein depletion reduces the level of Tor2, which makes the cell more robust against Torin 1 inhibition?

This is an interesting question, and gets to the root of why we performed the SGA analysis (Fig 7 and Fig S16). When we uncovered the link between RP gene loss and the TOR pathway we were surprised and wondered how these mutants could survive in the presence of Torin 1, so we decided to take a broad genetic approach and check the dependence on many different candidate pathways. We opted to test multiple pathways instead of focusing on a single gene like Tor2 because we suspected that many different pathways could be involved and we could take advantage of the awesome power of yeast genetics. The results of the SGA screen confirmed our suspicion and provides a rich source of information for pathways that compensate for RP insufficiency in fission yeast, and likely many of these will be evolutionarily conserved. We have rewritten this section in a way that we hope better highlights the rationale and the information gained, and have included a new Figure S16 that better illustrates the growth phenotypes on Torin 1 for each RP mutant. We think the exact mechanism by which RP gene loss affects the TOR pathway in different RP mutants is an interesting question for future study and is beyond the scope of this manuscript identifying regulators of rDNA spatial organization.

3) R-protein mutants which do not compensate the inhibition of the Tor pathway generate a similar cytokinetic effect as 60S r-protein mutants with “high” transcription phenotype. Please explain!

Our results suggest that the pmk1 pathway is involved in a compensatory response to RP gene deletion. Interestingly, the phenotype appears pervasive across RPL and RPS mutations and is not specific to RPL hits with Torin1 resistance. We speculate that either RPL loss has increased pmk1 activity relative to RPS stains, or that RPL loss generates Torin1 resistance due to pmk1 activity in combination with other unknown changes in signaling. Given the number of pathways that regulate ribosome biogenesis, it is likely that RPS and RPL insufficiency triggers different combinations or intensities of changes in signaling pathways. We highlight some of the known discrepancies in RPL and RPS phenotypes in the discussion, lines 509-514 page 24. However, we decided to remove the section on the cytokinetic defects in the double mutants because a full explanation will require more experiments, allowing us to focus on strengthening other parts of the story.
