# Supplementary material for: Regulators of rDNA array morphology in fission yeast
Source: PLoS Genet. 2024 Jul 5;20(7):e1011331. doi: 10.1371/journal.pgen.1011331 (PMC11253961; doi:10.1371/journal.pgen.1011331)
Supplement: Attachment — Submitted filename: rebuttal.docx [file pgen.1011331.s027.docx]

Dear Dr Gerton,
Dear Jen,
Please let us first apologize for the delay in getting back to you.
We have now received reports on your revised manuscript from three expert referees.
All three referees concur that a substantial amount of work has gone into the revision.
As you will read yourself, their recommendations are very contrasted.
Referee #2 is well satisfied and suggests only minor (mostly formatting) changes.
Referees #1 and #4 are still not truly convinced by several aspects.
An important and somehow shared criticism that remains is the lack of definitive demonstration that the GapR-GFP probe binds exclusively to transcriptionally induced overtwisted rDNA.
We really appreciate the amount of work you, and your team, have put into this submission, and we still believe your work has potential for PLOS GENETICS.
Therefore, we suggest you carefully review the points raised by reviewers #1 and #4.
If you believe you can address them satisfactorily and provide decisive information on the remaining critiques (or refute them efficiently, for example, because you don’t think they impact your work), then we would be happy to receive rapidly a revision. Otherwise, we suggest it is may better to transfer to another journal for the sake of efficacy and a timely publication of your work.
Kind regards
Denis

Reviewer's Responses to Questions

**Comments to the Authors:
Please note here if the review is uploaded as an attachment.**

Dear Reviewers and Editor-

We thank you for your attention to this much revised version of our manuscript. One shared criticism was “the lack of definitive demonstration that the GapR-GFP probe binds exclusively to transcriptionally induced overtwisted rDNA.” We hear this concern of reviewers 1 and 4 and appreciate the opportunity to respond. GapR is a bacterial protein. Previous publications have demonstrated how it binds to overtwisted DNA, a condition that is often a consequence of high levels of transcription. There is a crystal structure of the dimer of dimers and how it best accommodates a narrow DNA helix that is overtwisted. The mutant version we used binds to DNA more nonspecifically. Although GapR has these interesting structures and DNA binding properties, we do not attempt to infer any information regarding supercoiling or transcription from GapR binding in this manuscript. We demonstrate by ChIP that the full length version binds outside the transcribed rRNA genes. We demonstrate that its expression does not interfere with rDNA function. Once these properties are established, we employ it as a tool because it is a live marker of the rDNA that reveals morphology. This is a tool that has been lacking and was able to provide new insights. Proving that it binds exclusively to transcriptionally induced overtwisted rDNA is not relevant for this manuscript. We have included a sentence on page 12 in the manuscript to this effect.

In brief we have made the following editorial changes to address the comments of the reviewers:

1) We have corrected minor mistakes in figure labeling and sizing.
2) We have added a reference to support our observations regarding transcriptional assays based on 4TU incorporation, page 14, reference 29
3) We have added masks for nucleoli to the online data repository associated with this manuscript
4) We edited several supplemental figure legends for accuracy and clarity (S4, S5, S6, S11, S14)
5) We have added a statement to explicitly state that we have not used GapR to indicate overtwisting of DNA or transcription, page 12.

We hope the reviewers and Editor will now find our manuscript suitable for publication.

Reviewer #1: The authors have put considerable effort into revising their manuscript. It is intriguing and original. There still are problems with the primary data.

Regarding the data collection:
• The key fluorescent probe (GapR-GFP) is thought to bind “overtwisted DNA,” but is that especially relevant to rDNA ? and – if so – why ? It sounds as though the authors turned to GapR-GFP since staining with Hoechst was not reproducible, not because GapR-positivity implies that the DNA has some interpretable characteristic.

Please see the paragraph above, we do not use GapR to provide an interpretable characteristic of DNA.

• All experiments depend on the addition of the GapR-GFP inducer, estradiol, for 5-6 hr (as opposed to steady-state). Is it reasonable to think that the many conditions that they study do not affect the rate or stability of induction ? If induction is variable, certainly any measurements of signal intensity are questionable.

We agree that the induction will differ but all measurements are normalized to expression level, as noted in the section on page 11 when we describe how the imaging was performed, so this is not a concern. “To account for variability in GapR-GFP protein levels, the rDNA mean GFP intensity was normalized to the total nuclear mean GFP intensity.”

• The data depend critically on the authors ability to quantitate the “area occupied by rDNA.” On the basis of what is shown – although much of the analysis seems to have been automated and shows some “statistical significance” – it is not possible to know how accurate those measurements are. Some examples also seem to contradict their graphs. Thus, for “representative” wt cells in Figure 4E, the Nhp2 signal is obviously small by comparison to Figure 1A/B. Why is this ??

There seems to be concern over the automated methods. We feel this is the least biased way to quantify the data since it removes human bias, and also allowed us to quantify hundreds of cells and perform multiple replicates of each experiment. To address this concern we now provide examples of the masks which correspond to the “area occupied by rDNA” in our online data repository for review: Stowers Original Data Repository at <http://www.stowers.org/research/publications/libpb-2399>

The example in 4E has a small zone of high fluorescence (which may have been more obvious in a printout) but that is not the entire nucleolus and overall the size is similar. It’s worth noting that there is heterogeneity in a population of cells and that is why it’s important to quantify many cells in multiple replicates.

• The authors now assure us that cell cycle progression does not interfere with their measurements and that the data pertain to G2. Since they refer to “early” and “late” G2, these time intervals should be designated in Figure 2B/C.

Thankyou for this suggestion, we have now added these labels.

• The authors compare the performance of GapR to a mutant that is said not to bind to overtwisted DNA in Figure 1B. The mutant protein does not highlight the rDNA arrays but, as shown, it does primarily localize to the nucleolus. The authors offer no further comment, but the only DNA sequences that are known to be in the nucleolus are rDNA sequences. Might this “other” accumulation of GapR influence the estimates of “rDNA volume” ? This depends critically on the definition of the masks that were used for image analysis, but the reader has no way to know whether the masks were applied appropriately.

The GapR protein contains an NLS and therefore is expected to localize to the nucleus and nucleolus, exactly as observed. Oftentimes exogenous proteins or overexpressed proteins will concentrate more in the nucleolus. We now mention this in the manuscript (page 10). Masks are now provided in our online data repository for review: Stowers Original Data Repository at http://www.stowers.org/research/publications/libpb-2399

• The authors should compare their “hits” to those identified by Perrimon (their reference No. 11).

In the Perrimon reference, ribosomal proteins are not high confidence hits. This highlights the novelty of using a protein that marks the rDNA instead of a nucleolar marker.

Reviewer #2: The revised manuscript entitled " Regulators of rDNA morphology in fission yeast" by Cockrell et al., explore the ribosomal DNA structure using a bacterial protein, GapR, known to associate with overtwisted DNA.

Overall, this revised study is very interesting and provide some important insight on rDNA structure in vivo. This revised version is extensively modified, and improved. Some results and interpretation have been clarified, or removed. Most required experiments have been performed, or carefully addressed. Some minor corrections would be of interest.


Major critique:

none

Minor critiques:

Size and orientation of figures is not homogeneous. This is not the best to evaluate data.

Supplementary figure (.eps format) are not formatted, with variable size, resolution, length. This should be corrected.

Thankyou for pointing this out, we have corrected it.

Legend of supplementary figures need some final corrections:

In all figures, significant difference (0.01 or 0.05) should be highlighted.

Thank you for pointing this out, we have now applied and defined asterisks consistently across all figures

S4 fig : please indicate in figure legend quantification of what.

Thank you for pointing this out. We explained the quantifications in the text and in the legend for Figure 3 and stated the same method was used for all subsequent figures. We have now included a summary of this information in legend for figure S4 and S6.

S5 fig : “Glucose starvation causes complete transcription shut-off”. This conclusion appears over-stated without a simple control. The assay rely on uptake of 4-thiouracil pulse labelling. Please indicate how uptake of 4-thiouracil was evaluate in glucose starvation? Other studies supporting this conclusion using other assay would also be of interest.
We did not evaluate 4-thiouracil uptake in glucose starvation. We have changed the title of figure S5 to “4-thiouracil incorporation is not detected in glucose starvation”. However, this observation is consistent with the literature. We have added a reference to a study that evaluated the activity of RNA pol I, II and III during glucose starvation in yeast that concluded that transcription by RNA pol I and II ceased, page 14, reference 29. Importantly, different methods were used. https://www.sciencedirect.com/science/article/abs/pii/S0167478100000166

S7 fig. : 25 μg/μl Actinomcyin D (please correct spelling).
Thankyou for pointing this out, we have corrected it

Figure S11: Color used in the figure are not very clear. Yellow and gray are clearly explained . Why adding :”other colors indicate genes that did not validate for either rDNA parameter.” Which color (green, purple). Some, but not all background color are explained within figure. Please correct

Thank you for pointing this out. For simplicity, these nodes have been recolored white to represent that they did not validate for either rDNA volume or GFP intensity and a note has been added to the legend.

Legend of S14 Fig. is entitled :”RPL mutants with rDNA organization phenotypes do not have altered level of nascent RNA”, suggesting that more than one RPL mutant will be shown. Within figure, only rpl3001∆ is quantified. Please correct.

Thank you for pointing this out, we have corrected it

Idem for figure S15, mentioning “RPL mutants”, and showing rpl3602∆ and mrt4∆. Please clarify.
Thank you for pointing this out, we have edited the figure legend title to reflect that mrt4Δ is an assembly mutant.

Figure S16A, please correct spelling within figure (signaling pathay…)

Thank you for pointing this out, we have corrected it

Reviewer #4: The authors invested a significant amount of work to strengthen the interpretation of their results. Many issues were clearly addressed, but, unfortunately, an important concern remains the lack of a proof that GapR-GFP binds exclusively to transcriptionally induced overtwisted DNA in the different mutants - or physiological conditions. It is convincing that changes in the morphology of the nucleolus occur in the analyzed mutants and conditions, but this observation is not novel unless it is clearly shown that this is due to alteration of the rDNA topology in dependency of the transcriptional state.

1) It is important to convincingly show that GapR-binding correlates with alterations of rDNA topology and not with other expected or unpredictable consequences of nucleolar perturbances. Impairment of ribosome biogenesis by depletion of distinct r-proteins can result in drastic changes of rRNA processing, perturbation of the elongating Pol I machinery and/or R-loop formation. All these could provide a platform for (unspecific) GapR-binding. Analysis of a GapR-mutant that binds DNA nonspecifically cannot rule out these concerns. The authors must show at least one solid proof-of-principal-experiment that the DNA topology in the vicinity of GapR-GFP binding is indeed altered as a consequence of a transition to a different physiological state or in a mutant genetic background.

Please see the first paragraph. We do not use GapR to indicate topology or transition to a different physiological state (statement added to page 12), only as a visual marker of rDNA morphology.

2) The authors use A43-GFP as rDNA binding protein to underline the GapR-GFP result. A43 is part of the Pol I transcription machinery which is transiently associated with the ribosomal gene and might dissociate when ribosome biogenesis is impaired. It would be more straightforward to use a protein which tightly (constantly) binds to rDNA in areas not transcribed by Pol I. It is not analyzed in this study if r-protein depletion affects Pol I association with the rDNAs or the cellular protein level of Pol I subunits.

Finding an alternative RNA pol I factor that 1) binds to the rDNA in areas not transcribed by pol I, 2) binds constantly to the DNA throughout the cell cycle, and 3) can tolerate a fluorescent tag without impairing its function in rRNA synthesis, is challenging. We encountered this when we screened 4 alternative RNA pol I factors besides A43-BFP. GapR perfectly fulfills these 3 requirements, as we demonstrate in figures 1C and S3. Furthermore, GapR has the advantage that it is an exogenous protein without a function in the yeast genome.

3) To correlate GapR-binding effects and nucleolar size with Pol I transcription it is important to exclude RNA processing or degradation effects. Dot blot analysis of RNA isolated from cells treated for 5 min with 4-Thio-Uracil presented in this manuscript detects all kinds of RNAs. Depletion of r-proteins can result in rapid degradation of rRNAs and, therefore, dot blot analysis of 4-Thio-Uracil labeled newly synthesized RNA is probably not the best measure for Pol I transcriptional activity. Accordingly, it is important to visualize newly synthesized mature and premature rRNAs. This allows also to evaluate whether Pol I transcription and/or pre-rRNA processing is affected in the mutants and in

We agree that additional characterization of the rRNA transcripts could provide additional insight but we feel this is beyond the scope of the current manuscript. The results of our 4-TU assay for glucose starvation are consistent with a previous publication that used different methodology, now noted, Pag 14, reference 29.
